# Checklist for a complete chronic urticaria medical history: an easy tool

**DOI:** 10.1186/s40413-017-0165-0

**Published:** 2017-10-03

**Authors:** Ivan Cherrez-Ojeda, Karla Robles-Velasco, Pamela Bedoya-Riofrío, Peter Schmid-Grendelmeier, Sofía Cherrez, Florian Colbatzky, Ricardo Cardona, Pedro Barberan-Torres, Erick Calero, Annia Cherrez

**Affiliations:** 1 Universidad Espíritu Santo, Km. 2.5 vía La Puntilla, Código postal: 0901-952 Samborondón, Ecuador; 2 Respiralab, Respiralab Research Group, Guayaquil, Ecuador; 3 School of Medicine, University of Heidelberg, Heidelberg, Germany; 4 Allergy Unit, Department Of Dermatology, University Hospital of Zürich, Zürich, Switzerland; 5 Grupo de Alergología Clínica y Experimental, Universidad de Antioquia, Medellín, Colombia

**Keywords:** Chronic urticaria, Check list, Medical history, Chronic spontaneous Urticaria, Chronic inducible Urticaria

## Abstract

**Background:**

Existing guidelines do not offer a quick, efficient alternative to the patient’s recollection of relevant clinical features during anamnesis and physical examination for chronic urticaria (CU). This study aimed to identify specific items reflecting the main characteristics of CU that should be included in a comprehensive medical history for patients with CU. We also aimed to clarify possible eliciting factors for CU to support accurate diagnosis of the disease.

**Methods:**

A panel of postgraduate dermatologists conducted a literature search for relevant studies on CU using Medline, the Cochrane database, and PubMed.

**Results:**

We identified82 articles from which we drew a collection of items to inform development of an easy-to-use checklist and collection of items that should be included in a correct medical history. The final version of the checklist included42 items across two areas: essential clues for anamnesis and diagnosis of CU; and typical symptoms/parameters or characteristics according to subtype, etiology, and laboratory findings. Items included time of disease onset; duration, shape, size, color, and distribution of wheals; associated angioedema; atopy; and triggering factors.

**Conclusions:**

Our guide provides an easy-to-use tool to support clinicians to focus, orient themselves, and save time in medical consultations for CU, allowing better diagnosis and management of this disease.

**Electronic supplementary material:**

The online version of this article (doi:10.1186/s40413-017-0165-0) contains supplementary material, which is available to authorized users.

## Background

Urticaria is characterized by the development of wheals (hives), angioedema, or both [1]. Three common features of wheals are swelling and erythema, itching/burning sensations, and transient nature with the skin returning to a normal appearance within 1–24 h [2]. Angioedema is characterized by sudden localized, pronounced erythematous or skin-colored swelling of the lower dermis and subcutis, with frequent involvement below mucous membranes and sometimes pain rather than itching. Angioedema is slower to resolve than wheals, and can take up to 72 h [1, 3].

The prevalence of CU in the general population ranges from 0.5–5%, with the incidence of CU in Western countries estimated at 1.4% per year [1]. In Spain, CU ranks fourth among the allergic diseases after rhinitis, asthma, and drug allergy [4];the prevalence of CU is 0.6%, and is significantly higher in women than men. The average age of onset is 40 years [5]. A study in the United Kingdom showed that the prevalence of all forms of urticaria was 8.8% [4]. In Italy, the prevalence of chronic spontaneous urticaria (CSU) was 0.38% in 2013, with an incidence of 0.10–1.50 per 1000 person-years; female patients outnumbered males in both prevalence and incidence rates [6]. An Argentine guideline for urticaria and angioedema indicates 20% of the population have urticaria at some point in their life [7]. However, the point prevalence, cumulative prevalence, and lifetime prevalence of chronic urticaria (CU) in Latin America have not yet been established.

Urticaria is classified into two major types according to duration: acute urticaria (<6 weeks) and CU (≥6 weeks) [2]. For clinical purposes, CU is further classified into two subtypes: CSU and inducible urticaria (CINDU). CSU is characterized by the spontaneous appearance of wheals, angioedema, or both, resulting from unidentified causes and physiopathology that is not fully understood. Currently, the only identified aggravating factor is nonsteroidal anti-inflammatory drugs (NSAIDs). CSU also has spontaneous remission, making it difficult to prove cause-effect relationships.

There is a subgroup of CSU patients (40%) who has autoantibodies against the FcεRI, the high affinity receptor for IgE, that can stimulate and activate mast cells. The presence of these autoantibodies may be clinically important in a group of severely affected, treatment-resistant patients, where immunomodulatory treatments may be valuable. Patients with autoantibodies have no distinctive diagnostic clinical features. However, they do tend to have more severe urticaria [8].

In contrast, CINDU has known triggering stimuli, which can be classified as symptomatic dermographism, caused by cold, delayed pressure, sunlight, heat, vibration, cholinergic, contact, or water [1]. There is often an overlap between CSU and CINDU (e.g., a factitious component in CSU), and there is a tendency to sub-summarize both forms as CU [9].

We included urticarial vasculitis (UV) in this study. UV is not a form of CU, however is necessary to exclude it in patients with CU. In UV, the lesions are painful, burning, and tender, with plaques lasting for more than 24 h (sometimes up to 72 h). The wheals are associated with residual purpura or hyperpigmentation, and can have a central dark red or brown macule signifying underlying purpura and vasculitis. Other characteristics of UV are swelling, residual bruising, and edema from focused pressure; in such cases, skin biopsy would confirm or reject the diagnosis [10–13]. A study in Ecuador found that 10% of the population has UV [14].

This study aimed to determine a collection of items covering the main characteristics of CU that should be included in a comprehensive medical history to support thorough anamnesis and physical examination. We also evaluated possible eliciting factors of CU to aid accurate diagnosis of the disease and obtain comprehensive information on each patient that will then allow to select the best approach for treatment.

It is important to emphasize that this collection of items should not limit physical examination findings, which can vary and include features of other forms of urticaria; however, it provides a way to orient physicians in thinking about CU.

## Methods

For the purpose of this study, a panel of postgraduate dermatologists conducted a literature search. Inclusion criteria were: studies on the approach for patients with urticaria, anamnesis, physical examination, clinical features, characterization of lesions, and factors triggering CU; studies in English and Spanish; and articles published before 2016. Databases searched included Medline, the Cochrane database, and PubMed. Studies about the prevalence and treatment of CU were excluded.

The criterion used to identify relevant items was specific patterns or features of lesions described in the selected studies that correlated with the forms of CU. For example, localized hives appearing after a temperature change (cold or heat urticaria), and those induced by pressure (e.g., delayed pressure urticaria).

These items were included in the first draft and subsequently, a medical history was performed. A new revision and agreement between authors was done until the final version of the checklist was designed.

## Results

In total, 82 articles were selected and used to develop a 42-item checklist for taking a comprehensive medical history for CU. The guide and checklist are presented in Additional files 1 and 2. Checklist items covered two main areas:

1. Essential features for anamnesis and diagnosis of CU.
2. Typical symptoms/parameters or characteristics according to CU subtype, etiology, and laboratory findings.

Checklist items included:

1. Time of disease onset
  Very rapid (less than 2 h): CINDU: dermographism, 6–7 min; cold urticaria, 2–5 min; heat urticaria, a few minutes to hours [15]; aquagenic urticaria, 5–10 min [16]; urticaria from exercise, 6–25 min [17]; cholinergic urticaria, 6–25 min [17]; vibratory angioedema, 2–30 min; and solar urticaria, 2–15 min [18].
  More than 2 h: Pressure urticaria, 4–8 h [19].
2. Duration of wheals
  Less than 24 h: CINDU (<2 h), which includes dermographism (<30 min), aquagenic urticaria (<60 min) [16], solar urticaria (<60 min) [18], contact urticaria (2 h), and the majority of urticaria subtypes.
  More than 24 h: Pressure urticaria(24 h) [19]; UV(often 2–3 days, and may leave bruising) [2, 20].
3. Shape, size, color, and distribution of wheals
  Size 1–5 mm: cholinergic urticaria, heat urticaria, solar urticaria [21]. Greater than 5 mm: cold urticaria, chronic autoimmune urticaria. Coalescent: pressure urticaria [22]. Heat urticaria, solar and cold urticaria can come with small or large wheals depending on the area of skin that is exposed to the trigger.
  Shape: UV courses with petechiae/ecchymosis, livedo reticularis/Raynaud’s phenomenon, and sometimes blisters [23, 24].
  Color: erythematous (most hives); yellowish to red brown, urticaria pigmentosa (mastocytosis) [25]; erythematous with whitish halo, aquagenic urticaria [16] and CSU; purpuric, UV [23].
  Distribution: CINDU from pressure, solar and dermographic stimuli share a localized distribution [17]. Pressure and dermographic urticaria involve feet, hands, buttocks, and shoulders; cold, heat, and solar urticaria affect exposed areas; UV mostly affects the lower limbs [2, 17, 19]. Otherwise, cholinergic urticaria can be generalized (with a predilection for the trunk and upper extremities).Cold urticaria can be generalized if the entire skin is exposed to cold.
4. Onset symptoms
  Dermographic urticaria can sometimes begin with erythema and hives [26]. Cold urticaria presents with erythema, edema, and pruritus at onset. Pruritus, edema, and burning in pressure urticaria, cold urticaria and UV [17, 24].
5. Associated angioedema
  Through inherited deficiency of C1 esterase inhibitor, maybe localized or generalized; triggered by physical trauma, stress, drugs, or food; gastrointestinal compromise (angioedema) [27]. Typically, hereditary angioedema is not associated with urticarial skin lesions.
6. Associated subjective symptoms of lesions
  Itching, pain, and burning in pressure urticaria and UV. Burning in UV [23] and cold urticaria [28].
7. Family and personal history regarding urticaria and atopy
  Some reports mention a marked prevalence of atopy in CSU and CINDU, with the most common cases being allergic asthma, rhinitis, and contact allergy [29, 30].
8. Provoking factors for wheals
  Physical stimuli in CINDU: Cold (cold urticaria); heat (heat urticaria); sunlight (solar urticaria); water (aquagenic urticaria); pressure (pressure urticaria), machines that transmit vibration such as mixers or pruners (vibratory urticaria); rubbing (dermographic urticaria); physical exercise (cholinergic urticaria); and stress (CSU). Surgical implantations and events during surgery: implants, intrauterine contraceptive devices [31], surgical clips, metal prostheses [32], orthopedic implants [33], femoral prosthesis (e.g., Smith-Petersen Vitallium nail) [34]
  Infections: The association between infections and urticaria can be considered more direct in cases where urticaria disappears after treatment of infection. The role of infections in chronic urticaria as a causative agent in CU is still controversial however in a systematic review has been reported as an etiologic in approximately 6% of cases [35]. Identified etiologic agents are: bacteria (*Streptococcus* spp.*, Staphylococcus* spp.*, Yersinia, Helicobacter pylori*, *Treponema pallidum*); viruses(hepatitis B and C, cytomegalovirus, Epstein-Barr virus, norovirus); parasites,(*Blastocystishominis, Giardia lamblia* [adults]*, Toxocara canis* [children]*, Strongyloides stercoralis, Trichinella, Trichomonas vaginalis, Anisakis, Enterobius vermicularis* [pinworms]) [36]; and fungi (candidiasis, dermatophytosis) [35].
  Contact: latex, nickel, animals, steam cooking, perfumes, and pollens (rare cases reported) [1, 9].
  Drugs: NSAIDs can trigger CSU and angioedema [3, 37].
9. Diurnal and nocturnal variation
  Pruritus of urticaria in children and adults displays extensive temporal variability. It is worse nocturnally in chronic urticaria, often disturbing sleep, and is bothersome to a lesser extent upon awakening [38]. Recent findings suggest the circadian clock is an important regulatory component of local and systemic allergic reactions [39, 40].
10. Psychosomatic and psychiatric diseases
  Mainly anxiety and depression [41]. Patients with CU have higher scores in both the Beck Depression Inventory and the Beck Anxiety Inventory [42]. In addition, a reduction of productivity may be caused by sleep interference [43].
11. Gastric/intestinal problems
  Patients with symptoms of dyspepsia should undergo to a routine test to screen for *H. pylori*. Although *H. pylori* eradication has no discernible effects on CSU beyond that of standard CSU therapy, *H. pylori* eradication should only be initiated in accordance with currently accepted indications in its treatment guidelines [44].
12. Relationship to the menstrual cycle
  Menstrual Cycle is an aggravating factor in CSU. A rare form of urticaria is related to the menstrual cycle, appearing 7–10 days before menstruation [45, 46]. There are also pruritic urticarial papules and plaques of pregnancy occurring postpartum, related to skin stretching in the third trimester of pregnancy and characterized by the abrupt eruption of intensively pruritic papules (1–2 mm) that can coalesce to form plaques. These lesions spread over the distended abdomen [47].
13. Smoking habits (especially use of perfumed tobacco products or cannabis)
  Agents such as tobacco may aggravate CU [48].
14. Type of work
  Particularly vulnerable populations include healthcare workers, hairdressers, and food handlers [49]. Other vulnerable occupations are agricultural, dairy, and veterinary workers [50]. CU has also been reported during exposure to cyclic anhydrides (plumbers, packers, and painters) [51].
15. Previous diagnostic procedures/results
  Routine screening for malignancies in the diagnosis of underlying causes for urticaria is not suggested; however, if underlying causes are suspected, further research is necessary [1]. A prevalence of at least one anti-thyroid antibody has been described in 20% of patients with CSU, and is associated with more severe and prolonged urticaria progression. Urticaria has also been described as an initial finding for a patient with a carcinoid tumor [52]. Physicians should consider the possibility of neuroendocrine malignancies (specifically type I carcinoid tumors) when evaluating patients with rare urticaria presentations [52].

## Discussion

Physicians must promptly detect problems to successfully weigh the importance of findings in clinical settings and react appropriately. A thorough history and physical examination to identify all possible eliciting factors and causes of CU are important for diagnosis [53].

International guidelines for the diagnosis and management of CU are available, with most recommending diagnostic procedures when a doctor has a clinical suspicion of CU. These existing guidelines provide example recommendations to differentiate between CSU, CINDU, and other differential diagnoses, and to measure disease activity and impact. However, they do not offer a quick or efficient alternative to a patient’s recollection of relevant clinical features during anamnesis and physical examination. Most existing guidelines are general, and not particularly useful for clinical practice and informing oriented history-taking in the first doctor-patient meeting.

Physicians need an easy-to-use tool that helps them to quickly and easily diagnose this pathology. Therefore, this checklist has general and specific parameters ordered according to the sequence of a comprehensive clinical history. In addition, the checklist is able to be used by physicians independent of specialty (e.g., emergency rooms, primary care, internal medicine, and allergy and dermatology departments) without causing confusion as to the various differential diagnoses. The checklist will also contribute to the proper management of patients with CU from the start of clinical occurrence, achieving better control of the disease and reducing its social and economic impact. Delay in diagnosis statistically significantly increases severity, correspondingly increasing healthcare costs (specifically outpatient visit costs) and wages lost because of absences from work [8].

We developed an easy-to-use tool to support the early correct diagnosis and management of CU and facilitate healthcare providers/physicians’ diagnostic workup, clinical approach and allow to select the best approach for treatment in patients with CU.

To our knowledge, this is the first checklist available for diagnostic workup and management of patients with CU since 2010. In 2014, a clinical history of CU was published that provided a model for questions directed to patients suspected of this pathology [14]. However, that model did not cover specific features important in the clinical diagnosis of CU subtypes (such as color, characteristics of lesions, distribution of wheals, associated symptoms, and other possible etiologic agents), and did not include supporting evidence for each question as detailed in the present checklist [3, 14, 54].

In the last 30 years, attention has been focused on new ways to understand and manage urticaria. This includes the recent addition of novel drugs to the therapeutic arsenal, updating of clinical practice guidelines, and publication of pathophysiologic insights. Three decades ago, it would not have been possible to develop easy-to-use clinical guidance such as that developed here.

There are some limitations to our checklist. First, in CU, etiology is a major challenge for physicians because the frequency and relevance of infectious diseases vary among patient groups and geographical regions. We recommend further research to develop specific tools adapted to the context of each country. In addition, our study does not provide a “real-world” evaluation of the checklist or its impact. The present study did not intend to validate the role of our checklist in the medical history; consequently, face validity and reliability need to be established in a future study. Future studies should also evaluate the usefulness of this checklist. The initial version of the checklist was in Spanish, and it is also necessary to explore the usefulness of the checklist in other languages. A final limitation is that the checklist may be considered too long for some physicians. However, the length of the checklist is justified by relevant parameters that cannot be omitted from a clinical history for a patient with CU.

A strength of our study was that we performed an extended literature search to identify semiological and interrogatory characteristics that permitted our checklist to include all items necessary to collect comprehensive information from patients with CU. In a previous study, we found that knowledge of guideline recommendations among physicians who treat patients with urticaria in Ecuador was low [55]. The diagnostic workup and treatment of patients with CU was largely inconsistent with guideline recommendations in actual practice settings. The development of our checklist will help physicians to collect important patient medical data and facilitate workup and diagnosis. In future, it may also support collaboration in increasing knowledge about CU and its actual frequency, as data about the prevalence of CU are not available in Latin America [7].

## Conclusions

Urticaria consultations can be frustrating for both patients and physicians, because lesions may persist, factors that elicit urticaria are confusing, and there is a risk of misdiagnosis. The easy-to-use checklist developed in the present study should be adapted to the context of each country to reflect the most common CU etiology. Finally, this checklist will help focus and orient physicians, and save time in medical consultations, allowing physicians to perform better diagnosis and management of this disease.

## Additional files

Additional file 1: Chronic urticaria medical history (DOCX 2047 kb)

Additional file 2: Checklist for a complete chronic urticaria medical history (DOCX 1489 kb)

## Abbreviations

CINDU: Chronic Inducible urticaria
CSU: Chronic spontaneous urticaria
CU: Chronic Urticaria
NSAIDs: Non-steroidal anti-inflammatory drugs
UV: Urticarial vasculitis
