# Supplementary figures and images for: Checklist for a complete chronic urticaria medical history: an easy tool
Source: World Allergy Organ J. 2017 Oct 3;10(1):34. doi: 10.1186/s40413-017-0165-0 (PMC5625775; doi:10.1186/s40413-017-0165-0)

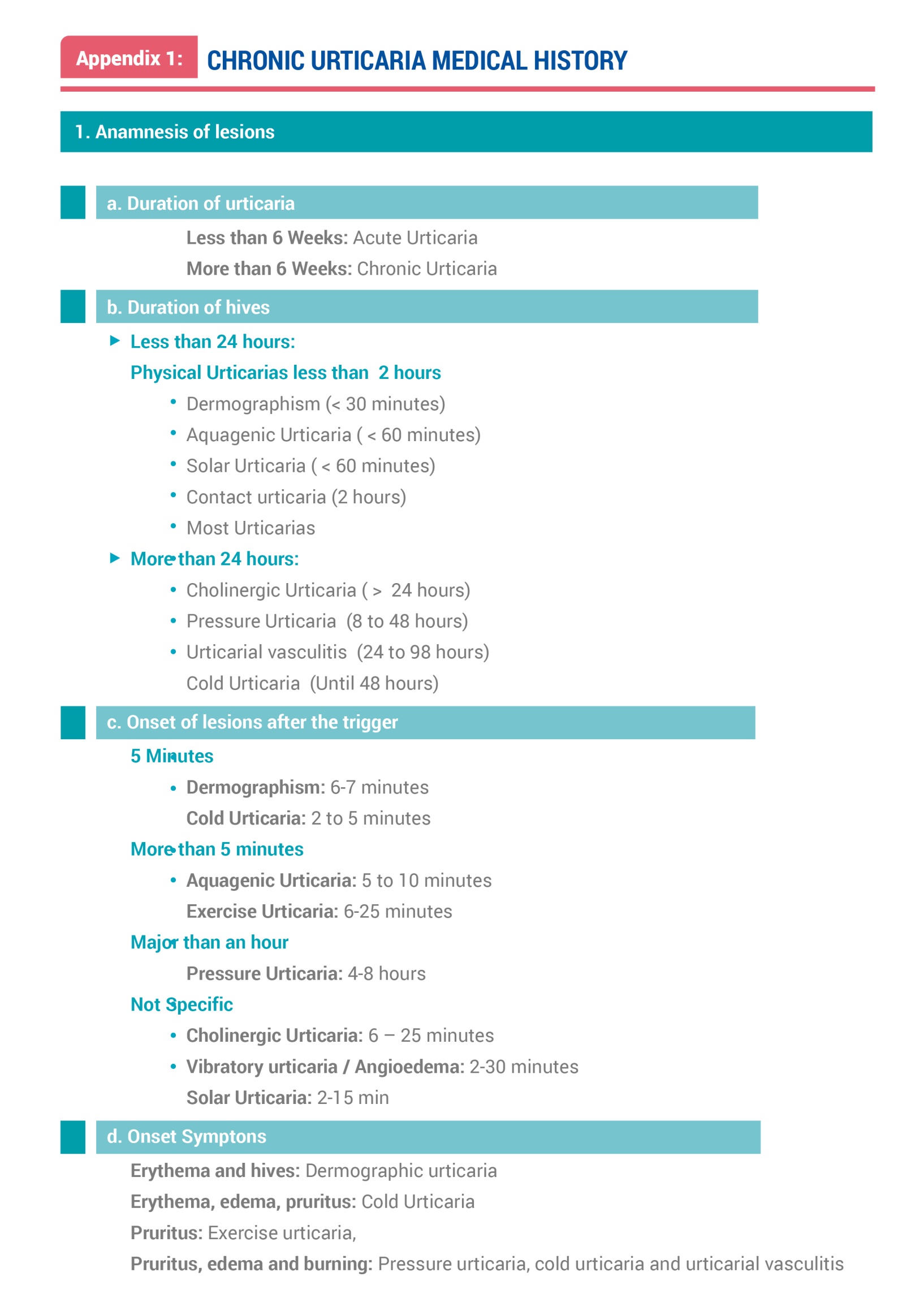


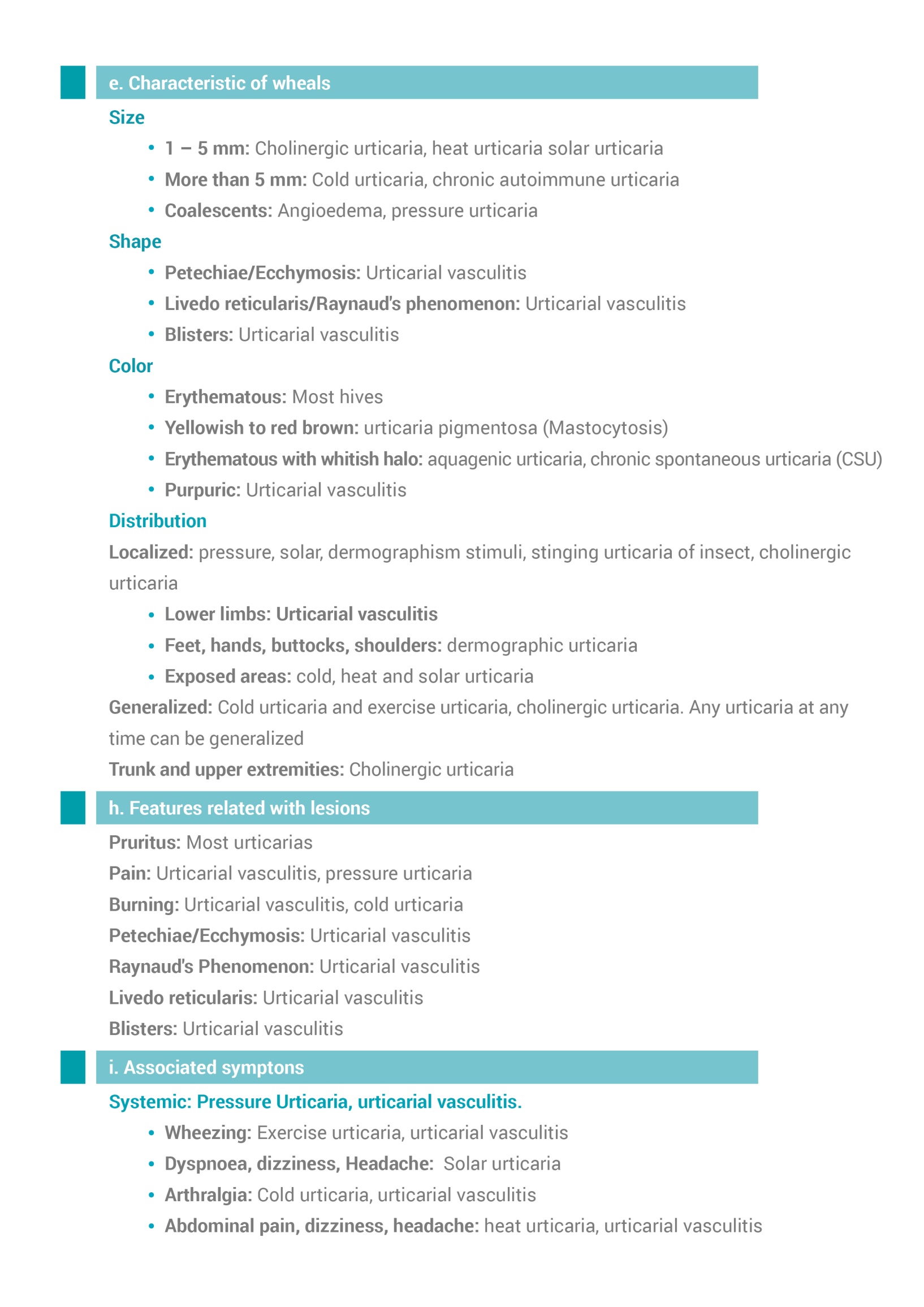


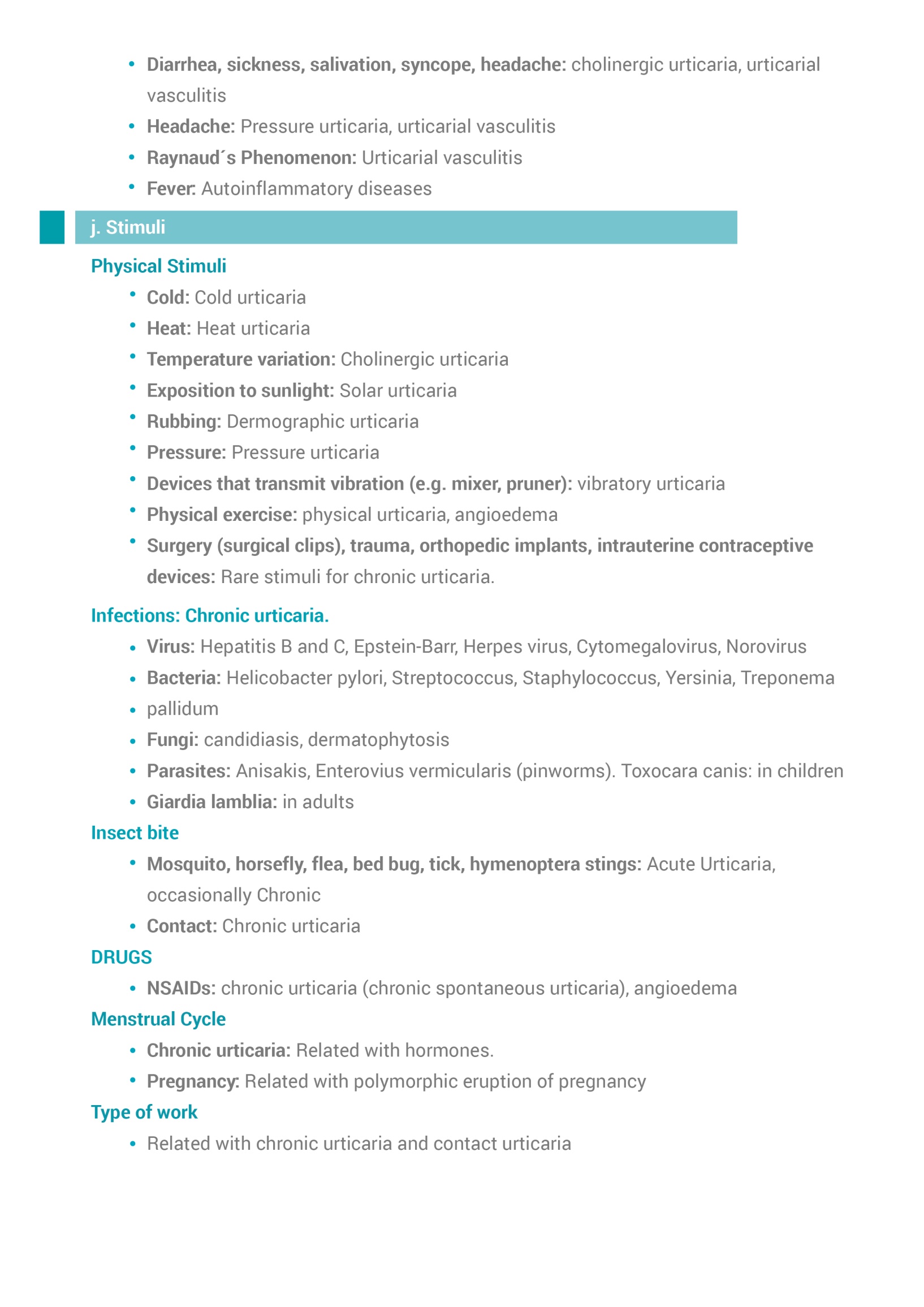


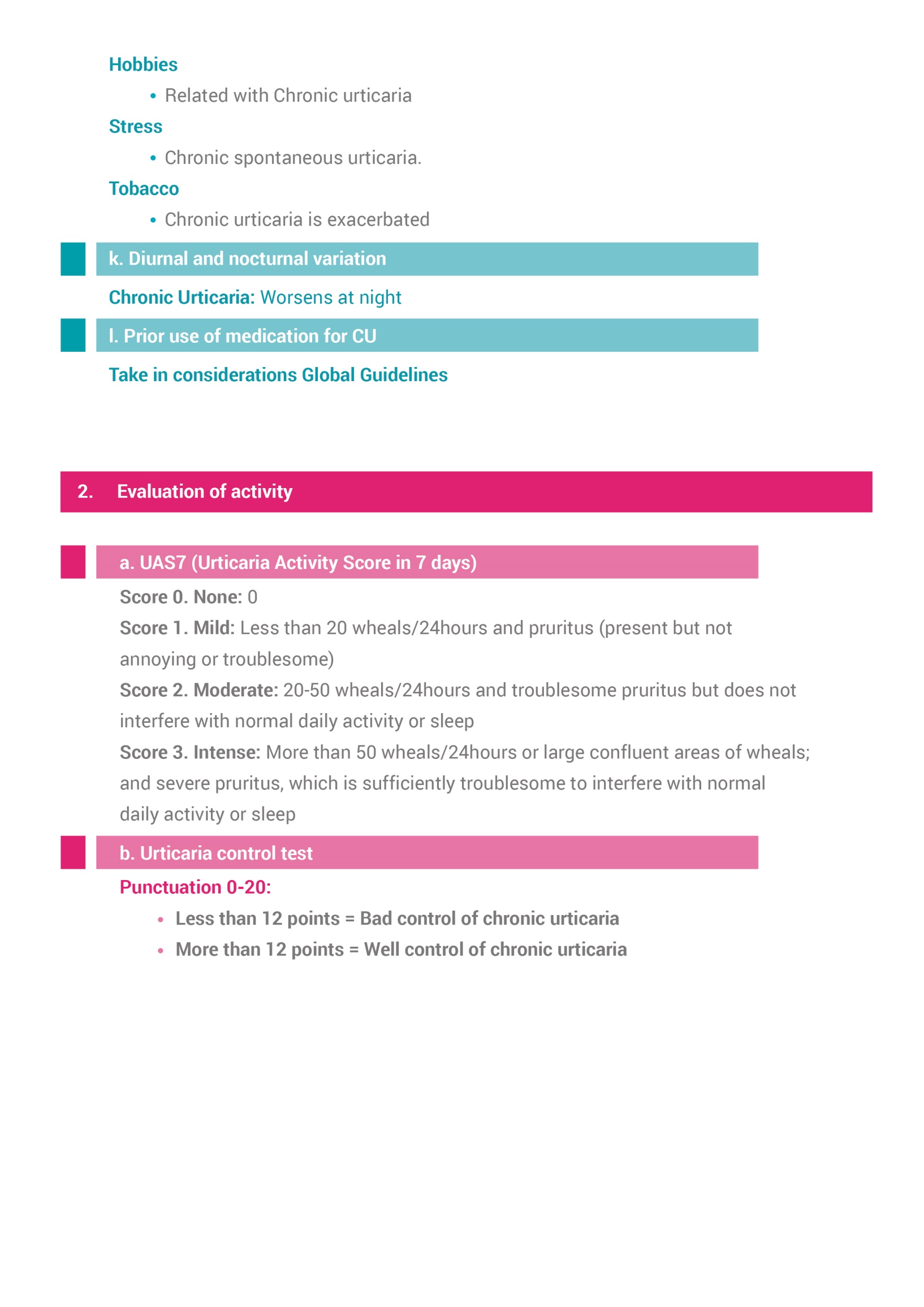


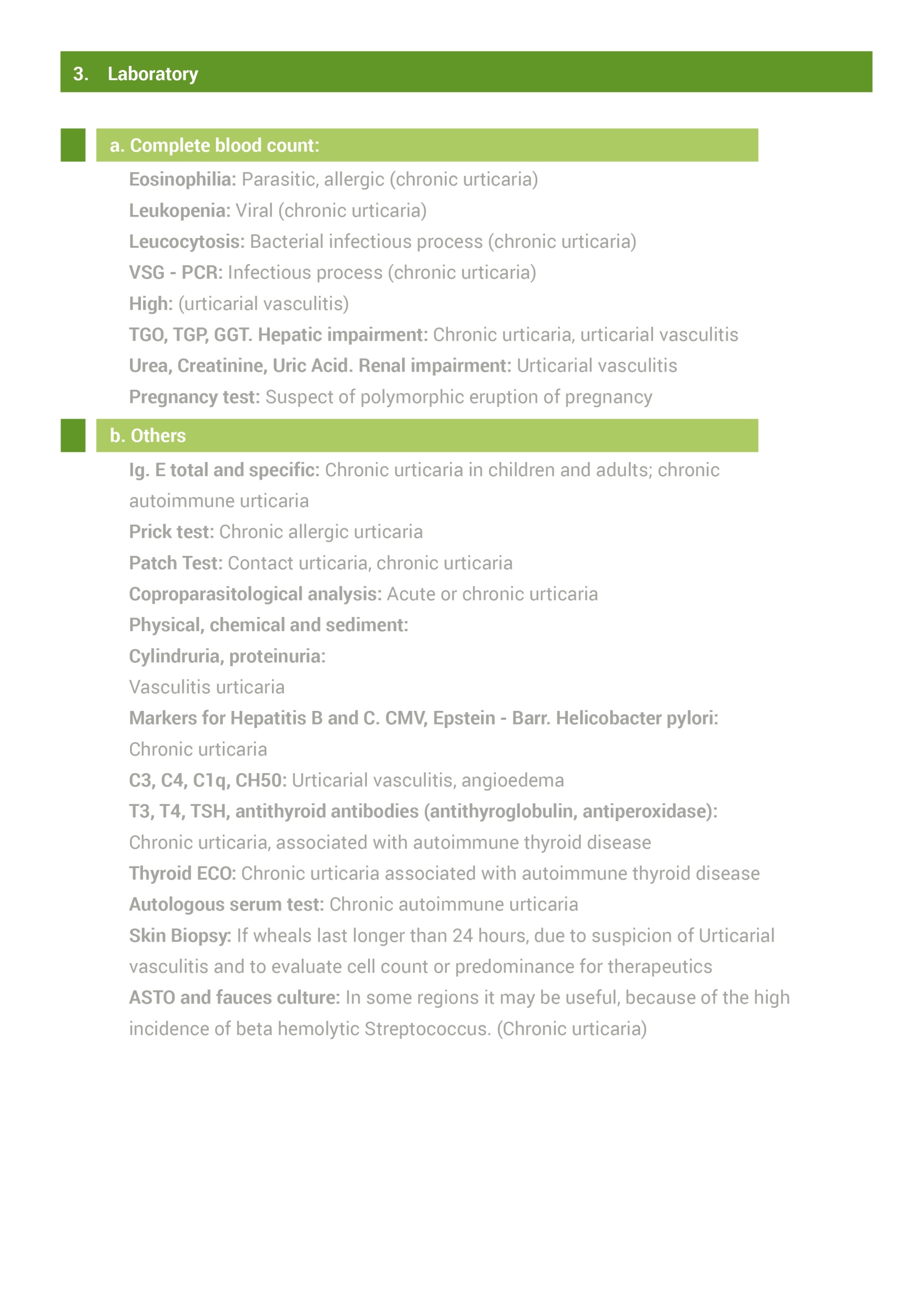

Supplement: Supplementary file 1 — Chronic urticaria medical history (DOCX 2047 kb) [file 40413_2017_165_MOESM1_ESM.docx]

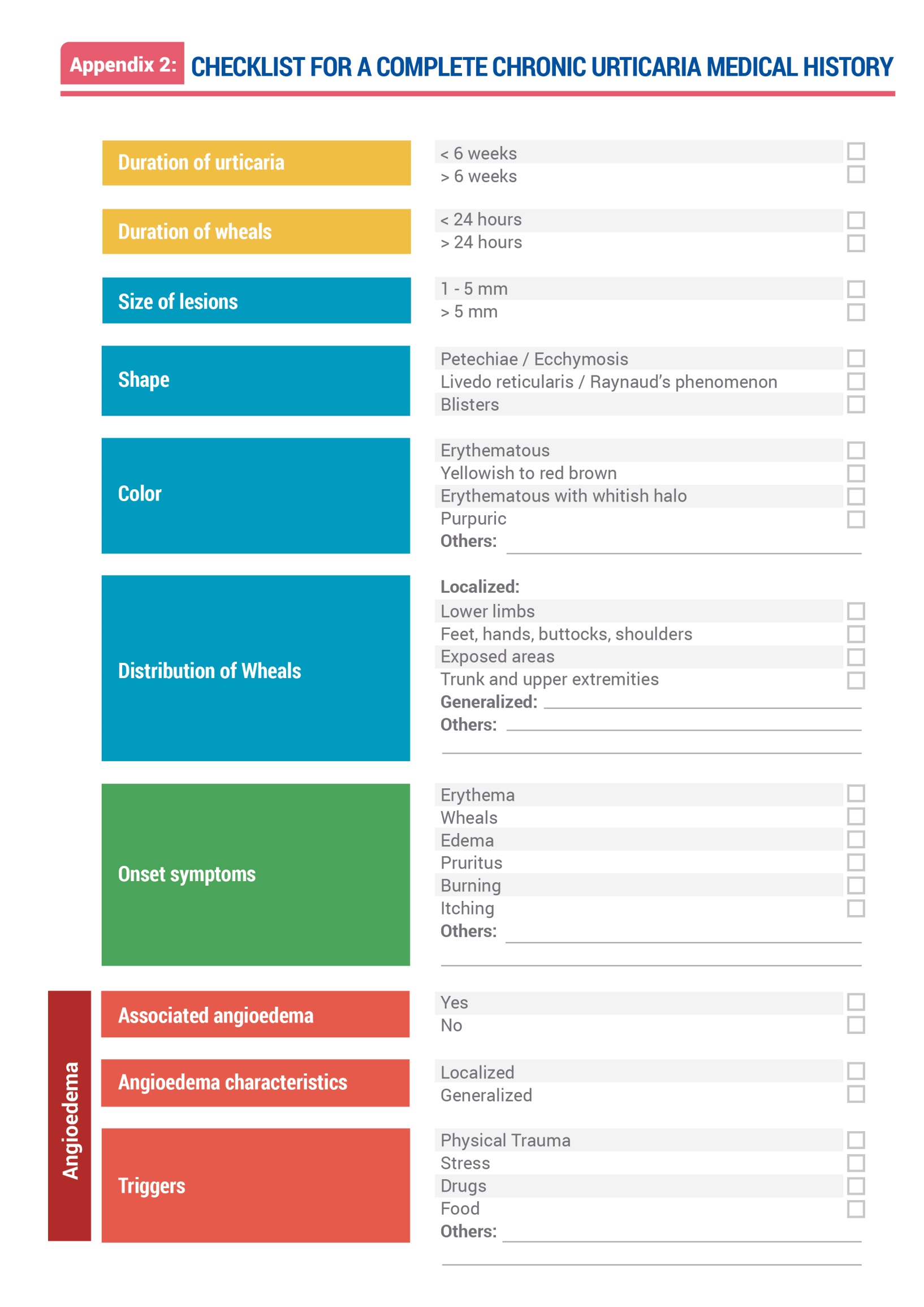


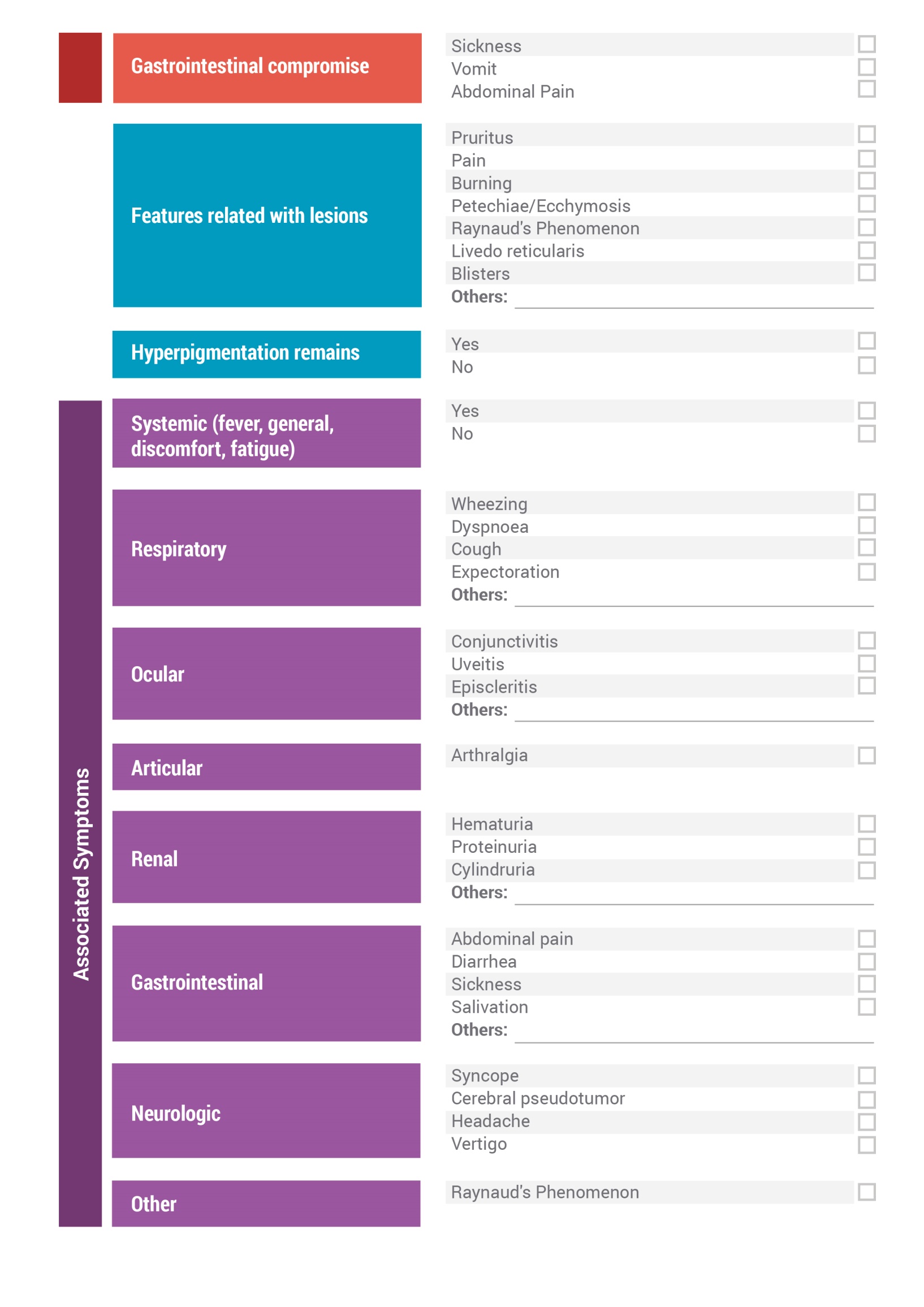


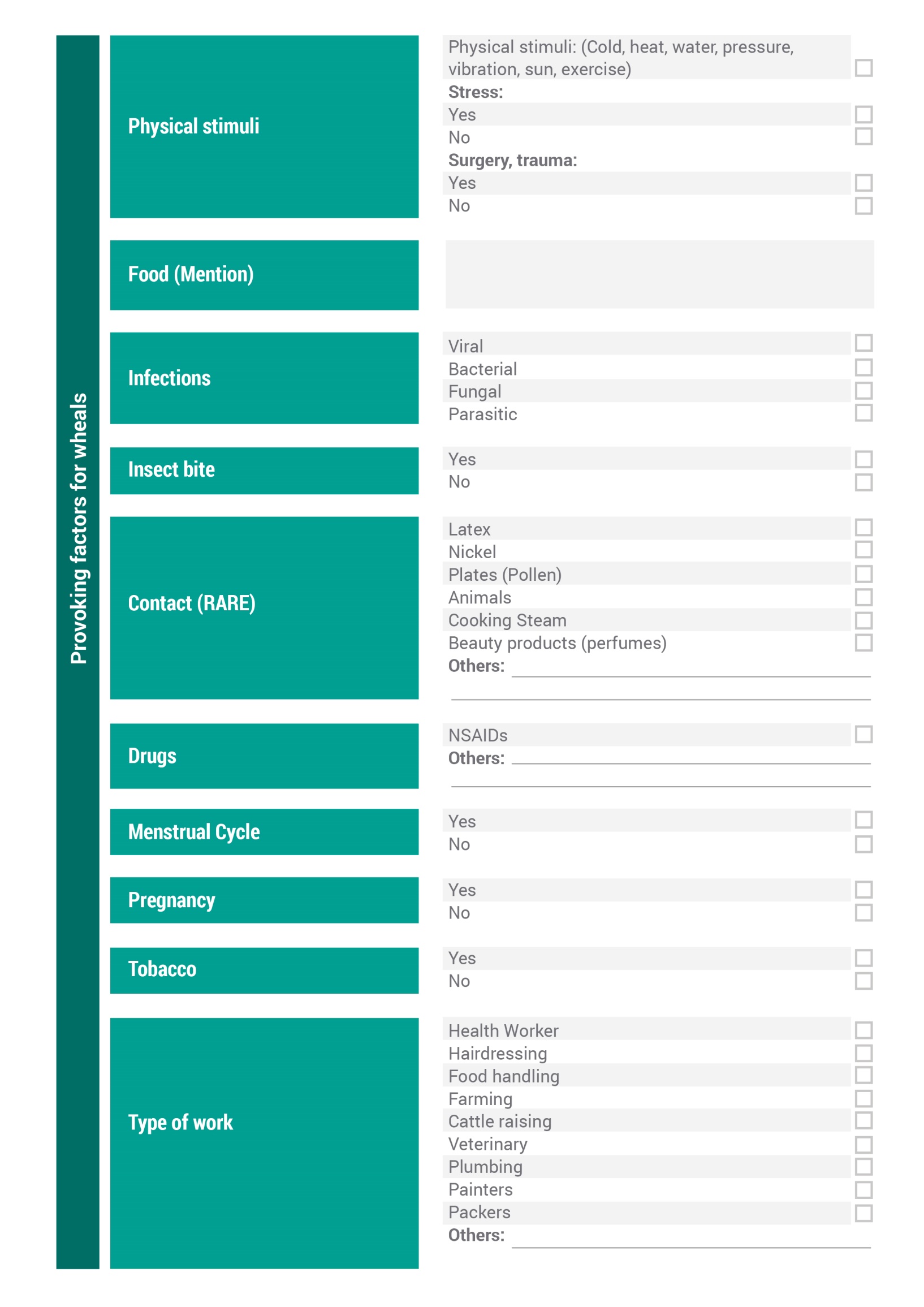


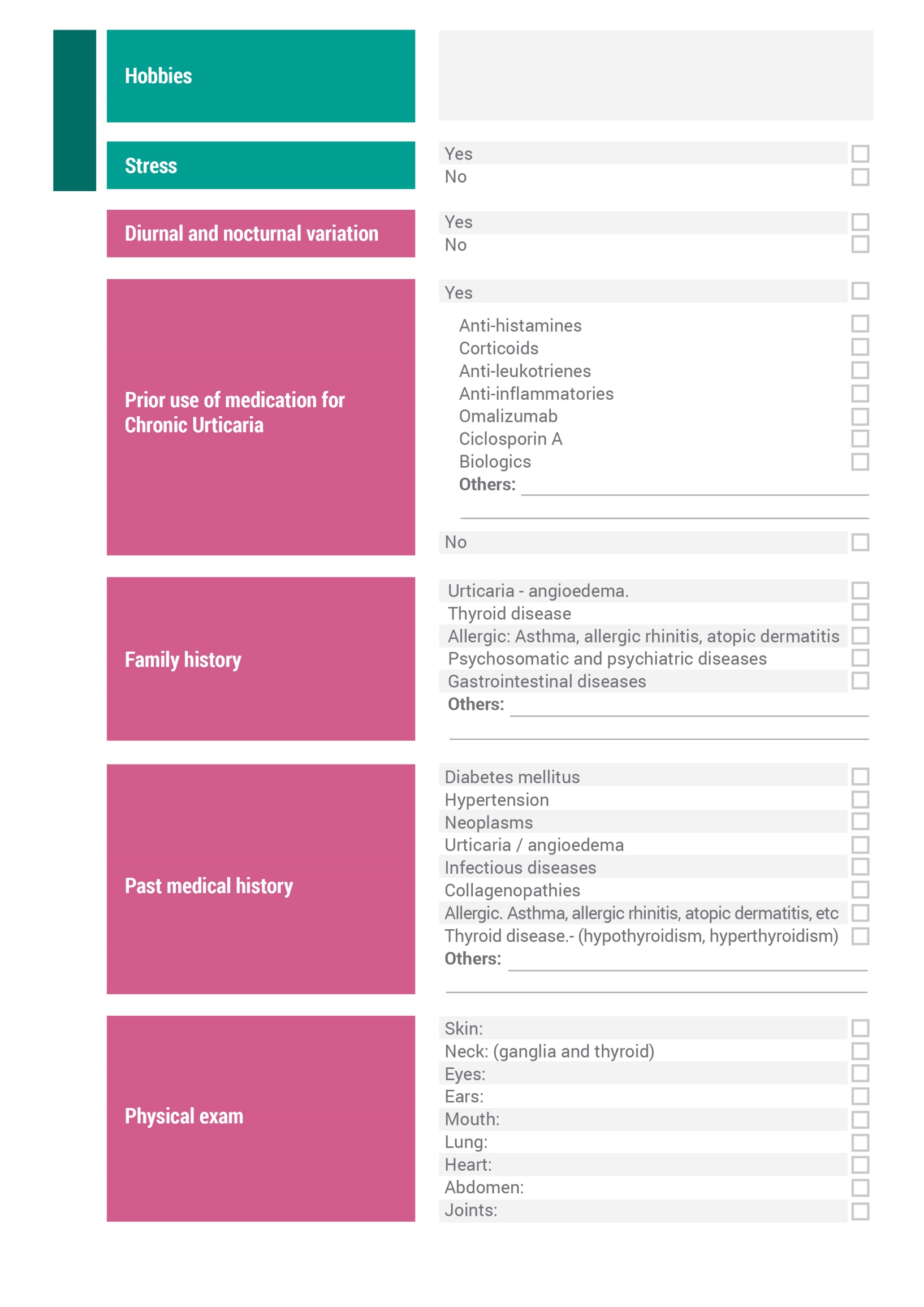

Supplement: Supplementary file 2 — Checklist for a complete chronic urticaria medical history (DOCX 1489 kb) [file 40413_2017_165_MOESM2_ESM.docx]
